# Development of a Novel Amplicon-Based Whole-Genome Sequencing Approach for Improved Surveillance of *Orthoebolavirus bundibugyoense* 2026

**DOI:** 10.3390/ijms27219501

**Published:** 2026-10-24

**Authors:** Martina Rueca, Eliana Specchiarello, Flavia Smoquina, Francesca Colavita, Cesare E. M. Gruber, Giovanni Chillemi, Fabrizio Maggi, Fabrizio Carletti

**Affiliations:** 1 Laboratory of Virology and Biosafety Laboratories, National Institute for Infectious Diseases Lazzaro Spallanzani-IRCCS, 00149 Rome, Italy; eliana.specchiarello@inmi.it (E.S.); flavia.smoquina@inmi.it (F.S.); francesca.colavita@inmi.it (F.C.); cesare.gruber@inmi.it (C.E.M.G.); fabrizio.maggi@inmi.it (F.M.); 2 European Union Public Health Reference Laboratories for Emerging, Rodent-Borne and Zoonotic Viral Pathogens (EURL-PH-ERZV); 3 Bioinformatics Research Unit in Infectious Diseases, National Institute for Infectious Diseases Lazzaro Spallanzani-IRCCS, 00149 Rome, Italy; giovanni.chillemi@inmi.it; 4 Department of Experimental Medicine, University of Rome “Tor Vergata”, 00133 Rome, Italy; 5 Head, Sequencing Unit, National Institute for Infectious Diseases Lazzaro Spallanzani-IRCCS, 00149 Rome, Italy; fabrizio.carletti@inmi.it

**Keywords:** ebolaviruses, bundibugyo, BDBV, whole genome sequencing

## Abstract

Bundibugyo virus (BDBV) remains one of the least genomically characterized filoviruses, partly due to the absence of primer schemes specifically optimized for its full genome amplification. Existing amplicon-based protocols developed for the Zaire Ebola virus or other orthoebolaviruses provide incomplete coverage when applied to BDBV, limiting genomic surveillance during outbreaks. In this study, we present a newly designed multiplex PCR primer scheme tailored to the genomic features of BDBV and optimized for sequencing on the Ion Torrent platform. The panel comprises 37 overlapping primer pairs spanning the entire ~19 kb BDBV genome, designed to minimize amplicon dropouts and ensure uniform coverage across regions with variable GC content and sequence divergence. Validation using BDBV RNA, derived from a diluted aliquot of a clinical specimen collected from a patient who tested positive for BDBV during the 2026 outbreak, demonstrated >99% genome recovery, high coverage uniformity, and robust performance even in low-titer samples. This represents, to the best of our knowledge, the first experimentally validated primer scheme specifically developed for BDBV. The protocol provides a reliable and accessible tool for genomic surveillance, outbreak investigation, and evolutionary studies, supporting improved diagnostic and public health responses to BDBV disease.

## 1. Introduction

Since May 2026, *Orthoebolavirus bundibugyoense* (BDBV), one of the six species within the genus *Orthoebolavirus*, has re-emerged in the Democratic Republic of the Congo (DRC), with sustained transmission and a rapidly increasing number of cases, followed by cross-border spread to Uganda and sporadic international exportation [1,2,3]. The outbreak has expanded considerably, prompting the World Health Organization (WHO) to declare a Public Health Emergency of International Concern [4]. As of 12 August 2026, a total of 4686 confirmed cases and 2186 deaths had been reported, including 4665 cases in the DRC, 20 in Uganda, and one imported case in France linked to the outbreak in the DRC, which represents the largest Ebola virus disease outbreak ever documented in the country [1]. This outbreak has highlighted the urgent need for robust genomic surveillance tools to support outbreak response and improve our understanding of BDBV epidemiology [5,6]. Whole-genome sequencing plays a critical role during filovirus outbreaks by enabling pathogen characterization, monitoring viral evolution, reconstructing transmission networks, and identifying genetic changes and the emergence of new variants that may affect diagnostics, therapeutics, or vaccine development. Rapid generation of high-quality genome sequences therefore represents a key component of public health preparedness and epidemic control. Although first identified during the 2007 outbreak in Uganda and associated with case fatality rates ranging from 25% to 50% [7], BDBV remains poorly characterized at the genomic level [8]. Most available sequencing protocols, including widely used schemes such as ARTIC, were developed for Zaire Ebola virus (*Orthoebolavirus zairense*) and Sudan Ebola virus (*Orthoebolavirus sudanense*) [9], and their performance on BDBV is suboptimal due to sequence divergence [10]. Amplicon-based sequencing has become a cornerstone of viral genomics, enabling rapid and cost-effective recovery of complete genomes even from low-titer samples. However, no primer scheme specifically designed for BDBV has been described to date, and no protocol has been optimized for Ion Torrent technology, which remains widely used in diagnostic laboratories.

In this study, we describe the design, optimization, and validation of a new primer scheme for full-genome amplification of BDBV. The scheme was developed through consensus sequence analysis, careful primer design, and practical optimization to ensure robust performance across all regions of the viral genome. We demonstrate that this protocol enables complete genome recovery with high uniformity and reliability.

## 2. Results

### 2.1. Primer Design and In Silico Genome Coverage

Alignment of representative BDBV genomes from the 2007, 2012, and 2026 outbreaks identified highly conserved genomic regions suitable for the development of an amplicon sequencing approach. Based on these analyses, a primer set generating of 37 overlapping amplicons was designed to cover the complete BDBV genome. The complete list of primers used is reported in Table 1. Amplicon sizes ranged from approximately 600 to 650 bp, with overlaps of 100–120 bp between adjacent amplicons, ensuring continuous genome coverage and redundancy across the sequencing targets (Table 1). In silico evaluation demonstrated broad compatibility of the primer set with BDBV lineages representative of the current 2026 outbreak in DRC and Uganda, and previous ones that occurred in 2007 and 2012, recorded epidemics included in the analysis. Although a limited number of primers (19 and 13 mismatches for 2007 and 2012 outbreak consensus sequences, respectively) showed overall one nucleotide mismatch per single primer relative to specific target sequences, these were located outside critical annealing regions and were not expected to significantly affect amplification efficiency (File S1).

### 2.2. Amplification Performance

Serial dilutions of BDBV-positive RNA coming from blood of a positive patient were subjected to multiplex RT-PCR amplification to determine the lowest RNA concentration at which the PCR amplification product remained suitable for downstream sequencing (Figure S1). Amplification was obtained for all primer pairs down to a dilution corresponding to 200 genome copies/µL. These findings demonstrate the robustness of the primer design and the ability of the multiplex assay to maintain efficient amplification down to a low titer of sample. Additionally, they also confirm the analytical suitability of the primer scheme for whole-genome amplification of BDBV, thus supporting its subsequent application for sequencing.

### 2.3. Sequencing Performance and Consensus Genome Generation

All 37 primer pairs successfully generated the expected amplification products without evidence of non-specific amplification or amplicon dropout. Amplicon coverage was highly uniform across the genome. Sequencing of the BDBV-positive sample generated 1,156,295 raw reads, with a mean read length of 228 bp. After quality filtering, 1,142,050 high-quality reads were retained, corresponding to ∼99% (98.87%) of the total reads generated. Of these, 801,551 reads mapped successfully to the BDBV DRC 2026 reference genome (Accession number: PP_006XCJJ.1), resulting in a mapping efficiency of ∼70% (70.19%). The mean genome coverage depth was approximately 9200×, with a median depth of 1900×. Coverage was evenly distributed throughout the genome, including highly variable *GP* gene regions that have historically proven difficult to amplify using cross-species filovirus primer schemes [11].

**Figure 1:**
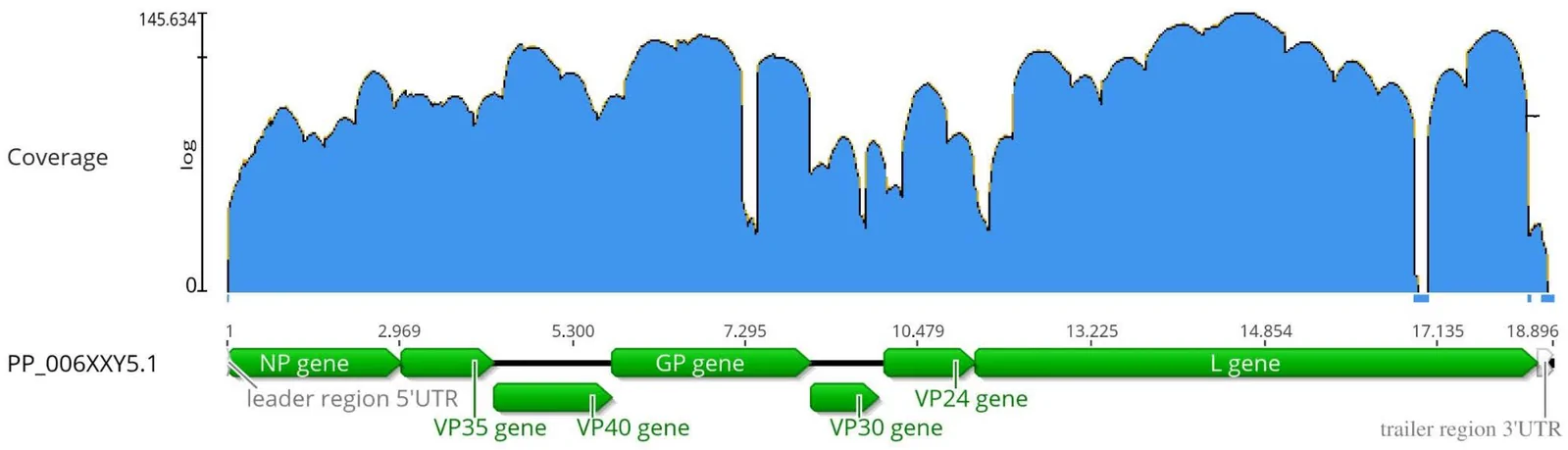
Plot of the genome coverage obtained using the novel amplicon-based BDBV whole-genome sequencing approach.

The sequencing approach was evaluated using a BDBV-positive clinical specimen with a real-time RT-PCR Ct value of 27. The analysis resulted in the reconstruction of 99.8% of the viral genome without the use of additional sequencing methods (Figure 1). More than 81% (81.25%) of genomic positions achieved a sequencing depth ≥ 100×, while ∼64% (64.47%) exceeded 500× depth. Coverage across all coding regions, including *NP*, *VP35*, *VP40*, *GP*, *VP30*, *VP24*, and *L* genes, was highly consistent, with no significant gaps observed. The few uncovered positions were located at the extreme genomic termini and did not affect coding sequence reconstruction. Regions previously reported as problematic for filovirus amplification, including portions of the *GP* gene and highly variable intergenic regions, were successfully resolved, resulting in complete recovery of biologically relevant genomic sequences.

## 3. Discussion

Unlike for other orthoebolaviruses (i.e., Zaire Ebola virus), optimized amplicon-based sequencing protocols for BDBV remain scarce because of the rarity of outbreaks and the limited availability of well-characterized clinical specimens. Nevertheless, previous studies have shown that whole-genome sequencing can provide valuable insights into BDBV evolution, transmission dynamics, and outbreak reconstruction. Analyses of viruses from both the 2012 DRC outbreak and the most recent outbreak support the hypothesis that the two outbreaks originated from separate spillover events [6,12,13], while experience gained during the West African Ebola epidemic demonstrated the broader value of real-time genomic surveillance for tracking transmission chains and viral evolution [14,15]. Despite these contributions, no dedicated workflow for routine BDBV whole-genome sequencing has been established thus far. Progress has been limited by the need for high-containment laboratories, poor access to suitable specimens, RNA degradation, variable viral loads, and the lack of standardized amplification strategies. To address these challenges, we developed an amplicon-based protocol specifically designed for BDBV whole-genome sequencing and optimized for Ion Torrent technology. The assay provided targeted amplification across the viral genome, consistent coverage, uniform read distribution, and complete genome reconstruction, providing cost-effective surveillance with substantially higher multiplexing capabilities compared with metagenomic sequencing approaches. Further, dilution experiments assessed performance at decreasing RNA concentrations, providing preliminary information on assay sensitivity and applicability to low-titer samples. Again, although validated on the Ion Torrent platform, the protocol could be adapted to other widely used sequencing technologies, including Illumina and Oxford Nanopore, following appropriate optimization. Additional studies involving a larger number of clinical specimens from different outbreaks will be required to fully evaluate assay performance across a broader range of sample types and viral loads. Although nearly complete genome reconstruction (99.8%) was achieved from a clinical specimen with a Ct value of 27, the availability of only one clinical sample represents a limitation of the study. Consequently, the clinical utility could not be established. Further evaluation using a larger number of clinical specimens would be required to assess the performance of the method in a clinical context in terms of characterizing its clinical sensitivity and analytical limit of detection. In conclusion, this study provides one of the first analytically optimized amplicon-based approaches specifically developed for BDBV whole-genome sequencing. The proposed workflow offers a practical framework for generating high-quality genomic data and may support future surveillance and outbreak-response activities involving this understudied orthoebolavirus.

## 4. Materials and Methods

### 4.1. Viral Sequences and Consensus Generation

Publicly available BDBV genome sequences were retrieved from GenBank (https://www.ncbi.nlm.nih.gov/nucleotide/; accessed on 22 July 2026) and from the Pathoplexus sequence sharing platform (https://pathoplexus.org/ebola-bdbv; accessed on 22 July 2026). Complete genomes from the 2007 (*n* = 6), 2012 (*n* = 19), 2026 Democratic Republic of the Congo (DRC; *n* = 106), and 2026 Uganda (*n* = 18) outbreaks were aligned separately using MAFFT v7.490 [16] to generate outbreak-specific consensus sequences. Consensus genomes were produced to enable an efficient and robust evaluation of the 37 primer pairs designed for full genome amplification: aligning each primer to a single consensus sequence per outbreak substantially reduces computational burden and simplifies mismatch inspection compared with aligning primers to all available genomes. Because some genomes from the 2026 Uganda outbreak exhibit small nucleotide differences (nt 4111–4151 of PP_XCJJ.1) relative to those from the contemporaneous DRC outbreak, the two sets were treated independently to ensure that primer performance was assessed against both region-specific viral lineages. The four resulting consensus sequences, representing the 2007, 2012, 2026 DRC, and 2026 Uganda outbreaks, were subsequently aligned to identify genomic regions suitable for primer design.

### 4.2. Primer Design

Primers were designed using Primer3Plus version 3.3.0 [17] and manually curated to ensure the following criteria: (i) melting temperatures between 58 °C and 62 °C; (ii) absence of secondary structures; (iii) minimal cross-dimer formation; and (iv) compatibility with Ion Torrent sequencing chemistry. The final scheme consisted of 37 primers covering the entire BDBV genome.

### 4.3. PCR Amplification and Sequencing

A BDBV RNA-positive RNA extract, obtained from a clinical sample collected from an infected patient and previously tested by Charité—Universitätsmedizin Berlin, was transferred to INMI Spallanzani within the framework of the European Reference Laboratory (EURL) network and used for the optimization and validation of the sequencing protocol. The patient provided consent to participate in this study.

Amplification was performed using a two-pool multiplex strategy comprising 18 primer pairs in Pool A and 19 primer pairs in Pool B. PCR cycling conditions were optimized to ensure balanced amplification across all target regions: 48 °C for 35 min; 95 °C for 15 min; 40 cycles of 94 °C for 30 s, 55 °C for 45 s, and 72 °C for 1 min; followed by a final extension at 72 °C for 10 min. Libraries were prepared using the Ion Plus Fragment Library Kit (ThermoFisher Scientific, Waltham, MA, USA) starting from 100 ng of amplified DNA after purification obtained by AMPure XP beads (Beckman Coulter, Indianapolis, IN, USA). Template preparation on the chip was performed with the Ion Chef instrument (ThermoFisher Scientific), and sequencing was carried out on the Ion Torrent S5 system (ThermoFisher Scientific) in order to obtain 1 million reads per sample.

### 4.4. Bioinformatic Analysis

Next-generation sequencing data were filtered, removing reads with a mean Phred score lower than 20, using Trimmomatic v.0.41[CG1.1] [18] software. Filtered reads were aligned to the BDBV genome PP_006XXY5.1 [19], using BWA v. 0.7.19 [20] software. Primer sequences were removed from mapped reads using iVar 1.4.2 [21] software. Finally, a whole-genome consensus sequence was generated, checked, and manually corrected with Geneious Prime 2025.1.3 (Dotmatics) software. Sequencing metrics including read quality, mapping statistics, genome coverage, and consensus completeness were calculated from the final alignment files.

## Figures and Tables

**Table 1:** Genomic localization of the 37 primers.

PRIMER ID	PRIMER SEQ. (5′-3′)	POSITION (PP_006XCJJ.1)	PRIMER ID	PRIMER SEQ. (5′-3′)	POSITION (PP_006XCJJ.1)
1F	ACTACGAGGAAGATTAAAGATTTTCC	2	19F	CGGAAATTTTGAGGCAGCCC	9103
1R	AGGTCTTGGATAGAGAATGTAGCAC	380	19R	ACAATTATCACTGGCGAATTGC	9463
2F	AGTTGACAACAGAGATACTAATCCA	275	20F	ACACCAGCATAAGTGAAATCTTGT	9399
2R	GACAGGCCTTTTCTCCGACA	894	20R	ACCAATCTAAAGGGGGCAACA	9782
3F	TGCTGCCTCAAGTGGAAAGA	774	21F	AAATAATTGGAAAACCAGAAAATCCC	9677
3R	TGCTTCTCGCAGTTGTTGGT	1427	21R	CCTACCTGTTGCCTTAGCCA	10,287
4F	CGTTTGCTCGTCTGCTGAAT	1292	22F	TAACGTGCCACACAGCTAGG	10,194
4R	CCTGGTCCCGTCCATTTCTC	1947	22R	GTGCCAATTTCGATACTGCTCA	10,812
5F	ATGCTGCAAGTGCTCCTGAT	1832	23F	GCAACTGAGCTTGAAGATGCT	10,714
5R	TCCTTCCCATCACTAGTGCTG	2442	23R	AGGAGTAATCCACAACTCAGGT	11,324
6F	CCCAGTCGGAGCAATCCATT	2333	24F	ACACCTAGAGAATTGCTGAATTGC	11,215
6R	AGCGTAGAAGAAATAGCTCCGAT	2990	24R	GCATCCTGCAAAACATAACTTACT	11,826
7F	CCCACAACAACAACCCCAAA	2853	25F	CGAACTTTATCCGGGGAGGG	11,761
7R	AGTTGCACGACCAGTTGTCA	3501	25R	ACCTTCTGAACCCAGTATCGAG	12,369
8F	GCAACGCATTACTGACCTGG	3385	26F	CACTCATTGCAGCATTGGCA	12,269
8R	GGTGATGGTGGAACTGGGC	3996	26R	AGGGAGGATGATCAAGGTGAT	12,902
9F	CAGCACCACCCGTAATCCAT	3930	27F	TGATAGCCAAGGGTCATGGT	12,777
9R	ACATCCGGTGCTGGAAAGTT	4514	27R	CGGTAACAAAACTGCTGCCTC	13,391
10F	CGCCAGAATACATGGAGGCT	4440	28F	AAGGAAAGCCTCTTGCACCA	13,321
10R	CCTCTTTTCCCGGTCTTCCC	5069	28R	CGCCATTTAAATATTGCTTCTTACCG	13,920
11F	GATCACTCAACCTCTCCCGG	4954	29F	CGTAGCAGCCAGTTTAGCCA	13,830
11R	TTGGGTCTATATGCTGCGGC	5562	29R	CCAGGGACATTCAATCCGCT	14,425
12F	CCATCCGACCCTCACCAAAA	5457	30F	TGCAAATGATCAACATGGACGA	14,336
12R	GTGTTGTTGTGAACTACGCCC	6076	30R	AGGGCATTGTTCGTACGATT	14,951
13F	CCCCGTGAACGCTTCAGAAA	5999	31F	TGGGCACACATACTAGAGGGA	14,869
13R	GTGTGGTAGTAGCTGGATGGG	6595	31R	GGCAACCGCAAAATTGATTACA	15,471
14F	GGCTTTCTTGATCCTCCCCA	6514	32F	ACGAGATTGGTGGTGTCGAC	15,385
14R	TTGGTAGTTCGTGGTGGCTG	7122	32R	CCTCAATGAGCGATGTGGCA	16,022
15F	GTTCAAGTGCGAGACCTCCA	7021	33F	TATAGTTTCGGCGCCATCGT	15,913
15R	TTTGCTAGCTGCCTCAACCC	7639	33R	TTGGTGGGTTTGAGGCACAT	16,536
16F	GATACCTTACTTCGGGCCCG	7563	34F	TTGGAGGAGCCGTCACAAAG	16,443
16R	GTCAGGTCATCTGGCTGTTCT	8170	34R	TAAGGCACCTGACCCTTCTC	17,024
17F	TGCTTCTGCTTCCTATAGTTAATACA	8148	35F	TGTCGCTTTACAGGGGTTGT	16,927
17R	GCTGTCACACAAAAATCCTTTCC	8673	35R	TAGCATGACCCTGATGAGGC	17,559
18F	CGAGTCCCGACTGTGTTTCA	8591	36F	TGCCCCTTTATTCGGATCCG	17,451
18R	GCACTAGCAGCCTTAATCCTGA	9221	36R	TCTGAGTCTTGCATACGGGT	18,069
			37F	AGAACTCAGAACACTTCCTGACT	17,964
			37R	ACTCAACAACTAACAACTGAGACT	18,617

## Data Availability

The data that support the findings of this study are available from the corresponding author upon reasonable request.

## Supplementary Materials

The following supporting information can be downloaded at https://www.mdpi.com/article/10.3390/ijms27219501/s1.

## References

[1] World Health Organization Disease Outbreak News; Bundibugyo Virus Disease, Democratic Republic of the Congo (The) and Uganda. https://www.who.int/emergencies/disease-outbreak-news/item/2026-DON602.

[2] Pereira M.R. (2026). Ebola disease caused by Bundibugyo virus, Democratic Republic of the Congo and Uganda. Am. J. Transplant..

[3] Zhang Y., Ren J., Ma X., He X. (2026). The 2026 Bundibugyo virus disease outbreak in the Democratic Republic of the Congo: Virology, epidemiology, continental response, and research priorities. Infect. Med..

[4] https://www.who.int/news/item/17-05-2026-epidemic-of-ebola-disease-in-the-democratic-republic-of-the-congo-and-uganda-determined-a-public-health-emergency-of-international-concern.

[5] Ayitewala A., Amuri-Aziza A., Nsawotebba A., Wawina-Bokalanga T., Ssewanyana I., Nabadda S., Kinganda-Lusamaki E., Kanyerezi S., Zalwango V.N., Mukadi-Bamuleka D. (2026). Bundibugyo ebolavirus from the 2026 Ebola outbreak in Uganda and DR Congo: A new variant. Lancet.

[6] Amuri-Aziza A., Luakanda-Ndelemo G., Ayitewala A., Jansen D., Adroba-Tandele P., Nsawotebba A., Kinganda-Lusamaki E., Kanyerezi S., Kaleebu P., Ngandu C. (2026). Emergence of a Bundibugyo virus variant in the 2026 outbreak in the Democratic Republic of the Congo and Uganda. Nat. Med..

[7] Boatemaa A., Osei B., Forkuo G.O. (2026). Mapping the global evidence base of bundibugyo ebolavirus disease: A systematic scoping review of research gaps and preparedness priorities. BMC Infect. Dis..

[8] Bongiovanni A., Binetti E., Colavita F., Mussetto I., Scorzolini L., Lalle E., Antinori A., Girardi E., Maggi F., Nicastri E. (2026). Bundibugyo virus disease: Transmission dynamics, infectiousness and viral persistence. Int. J. Infect. Dis..

[9] Quick J. (2019). Ebola Virus Sequencing Protocol. Protocols.io. https://www.protocols.io/view/ebola-virus-sequencing-protocol-e6nvw9p7dgmk/v1.

[10] Cheng N., Ye R.-Z., Li Y.-Y., Kargbo K.B., Ren L.-L., Cao W.-C. (2025). Global distribution and genetic diversity of orthoebolaviruses: Mapping and evolutionary analysis. One Health.

[11] Di Paola N., Sanchez-Lockhart M., Zeng X., Kuhn J.H., Palacios G. (2020). Viral genomics in Ebola virus research. Nat. Rev. Microbiol..

[12] Kainulainen M.H., Nichol S.T., Albariño C.G., Spiropoulou C.F. (2017). Rapid Determination of Ebolavirus Infectivity in Clinical Samples Using a Novel Reporter Cell Line. J. Infect. Dis..

[13] Hulseberg C.E., Kumar R., Di Paola N., Larson P., Nagle E.R., Richardson J., Hanson J., Wauquier N., Fair J.N., Makuwa M. (2021). Molecular analysis of the 2012 Bundibugyo virus disease outbreak. Cell Rep. Med..

[14] Gire S.K., Goba A., Andersen K.G., Sealfon R.S.G., Park D.J., Kanneh L., Jalloh S., Momoh M., Fullah M., Dudas G. (2014). Genomic surveillance elucidates Ebola virus origin and transmission during the 2014 outbreak. Science.

[15] Quick J., Loman N.J., Duraffour S., Simpson J.T., Severi E., Cowley L., Bore J.A., Koundouno R., Dudas G., Mikhail A. (2016). Real-time, portable genome sequencing for Ebola surveillance. Nature.

[16] Katoh K., Standley D.M. (2013). MAFFT multiple sequence alignment software version 7: Improvements in performance and usability. Mol. Biol. Evol..

[17] Untergasser A., Cutcutache I., Koressaar T., Ye J., Faircloth B.C., Remm M., Rozen S.G. (2012). Primer3—New capabilities and interfaces. Nucleic Acids Res..

[18] Bolger A.M., Lohse M., Usadel B. (2014). Trimmomatic: A flexible trimmer for Illumina sequence data. Bioinformatics.

[19] Best T.D., Melchert J., Mauno T., Zaki N.W., Veith T., Bleicker T., Jones T.C., Drosten C., Corman V.M. Democratic_Republic_of_the_Congo/PP_006XXY5.1/2026-05-20. https://pathoplexus.org/seq/PP_006XXY5.1.

[20] Li H., Durbin R. (2009). Fast and accurate short read alignment with Burrows-Wheeler transform. Bioinformatics.

[21] Grubaugh N.D., Gangavarapu K., Quick J., Matteson N.L., De Jesus J.G., Main B.J., Tan A.L., Paul L.M., Brackney D.E., Grewal S. (2019). An amplicon-based sequencing framework for accurately measuring intrahost virus diversity using PrimalSeq and iVar. Genome Biol..

